# Comment on “Orthorhombic Symmetry and Anisotropic Properties of Rutile TiO_2_”

**DOI:** 10.1021/acs.jpcc.5c00048

**Published:** 2025-06-04

**Authors:** Ilaria Tomei, Claudio Goletti

**Affiliations:** Physics Department, University of Rome Tor Vergata, Via della ricerca scientifica 1, 00133 Rome, Italy

In an article by Szwacki et al.^1^ recently published in *J. Phys. Chem C*, the authors reported new theoretical calculations and experimental data and concluded that rutile TiO_2_, in contrast to what is commonly believed, does not have a tetragonal symmetry but rather an orthorhombic symmetry. By pseudopotential plane-wave-based density functional theory (DFT) calculations, the *r*_*ab*_ parameter—that is, the relative difference of lattice parameters *a* and *b*—is computed, obtaining an inequivalence on the order of 1 × 10^–3^. The consequent anisotropy of the refraction index values *n*_*a*_ and *n*_*b*_ in the transparency region of TiO_2_ should then produce a birefringence effect in a beam of light linearly polarized, properly oriented and impinging at normal incidence on the (001) surface of a rutile TiO_2_ sample (see Figure 1, right panel). In particular, the polarization plane of the beam outgoing after passing *through the sample thickness* should rotate with respect to the polarization plane *before* the sample. This is what happens in a photo-elastic-modulator (PEM),^2^ rotating the linear polarization plane of light in reflectance anisotropy spectroscopy (RAS),^3^ a modulation optical spectroscopy widely used to characterize clean surfaces in ultrahigh vacuum conditions^4^ and in liquid,^5^ to investigate organic layers,^6^ low dimensional systems,^7^ and recently strain-engineered GaAsBi alloys.^8^

**Figure 1:**
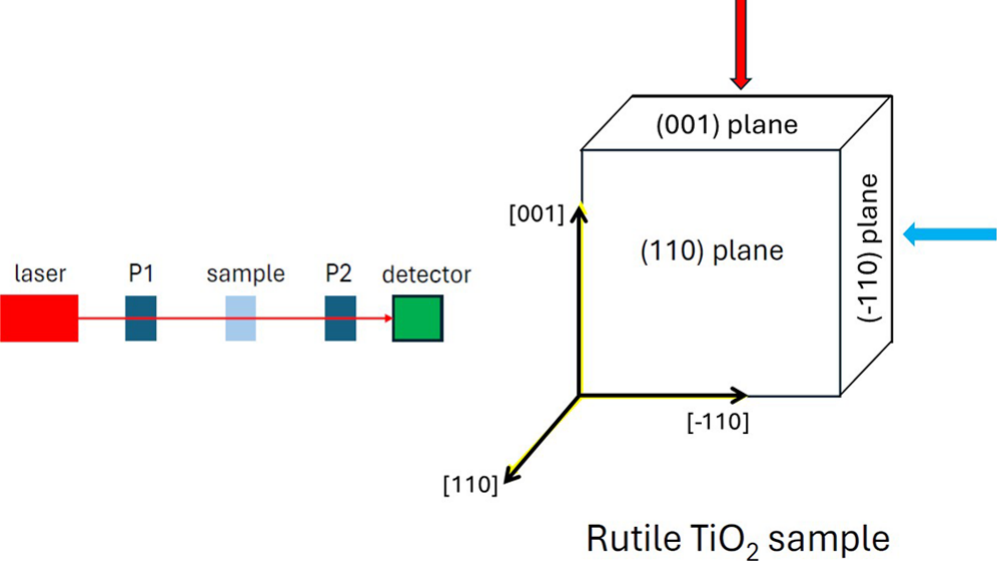
(Left panel) Sketch of the experimental apparatus. (Right panel) Orientation of the exposed polished planes in the TiO_2_ sample. The axes reported in figure are parallel to the [110] direction, to the [1̅10] direction and to the [001] direction. The (110) face has 5 mm × 5 mm^2^ sides, with a thickness of 0.5 mm.

We have performed an experiment to investigate the existence of an optical anisotropy between the two axes *a* and *b* of the TiO_2_ basis, in particular, measuring, if any, the birefringence effect associated with such a difference. The sample (S) (rutile TiO_2_, from PI-KEM, 5 mm × 5 mm × 0.5 mm) exposed polished oriented planes: (110), (001) and (1̅10), as shown in the right panel of Figure 1.

The beam of an He–Ne laser (λ = 6328 Å) has been linearly polarized by a Glan–Taylor polarizer (P1, Melles-Griot, extinction ratio better than 10^–5^). The beam was then directed onto a second polarizer (P2, identical to P1), the axis of which was set perpendicular to the axis of P1. No light (within the experimental accuracy) was detected by the detector (Si phototransistor, Hamamatsu) in this configuration. In a second step, the sample S was inserted between P1 and P2, thus measuring the transmitted light (Figure 1, left panel). The axis of P1 was carefully oriented with respect to the two main symmetry axes of the chosen plane of the sample (angle accuracy Δθ = 5 × 10^–4^ rad, namely 0.03°), that is perpendicular to the propagation direction of light. Great attention was used to align and orient the sample, to avoid inducing birefringence by an inaccurate position. We have chosen two different experimental configurations, depending upon the orientation of sample S (Figure 1, right panel):

(A) the laser beam shining at normal incidence onto the (001) plane of S, with the two sides parallel to the [110] and [1̅10] directions (see the red arrow in Figure 1). In this case, light was linearly polarized parallel to the [110] direction, thus resulting at 45° with respect to the *a* and *b* axes of the TiO_2_ basis;
(B) the laser beam shining at normal incidence onto the (1̅10) plane of S, with the two sides parallel to the [001] and [110] directions (see the cyan arrow in Figure 1). In this case, light was linearly polarized at 45° with respect to the [001] direction.

In Figure 2, we present the intensity of the light at 6328 Å measured by the detector after rotating the second polarizer P2 in an interval centered on the angle θ_0_ where the axes of P1 and P2 were crossed: (i) without S (curve “nosample”, blue); (ii) with S, in configuration A (curve “sample”, red); (iii) with S, in configuration B (cyan curve).

**Figure 2:**
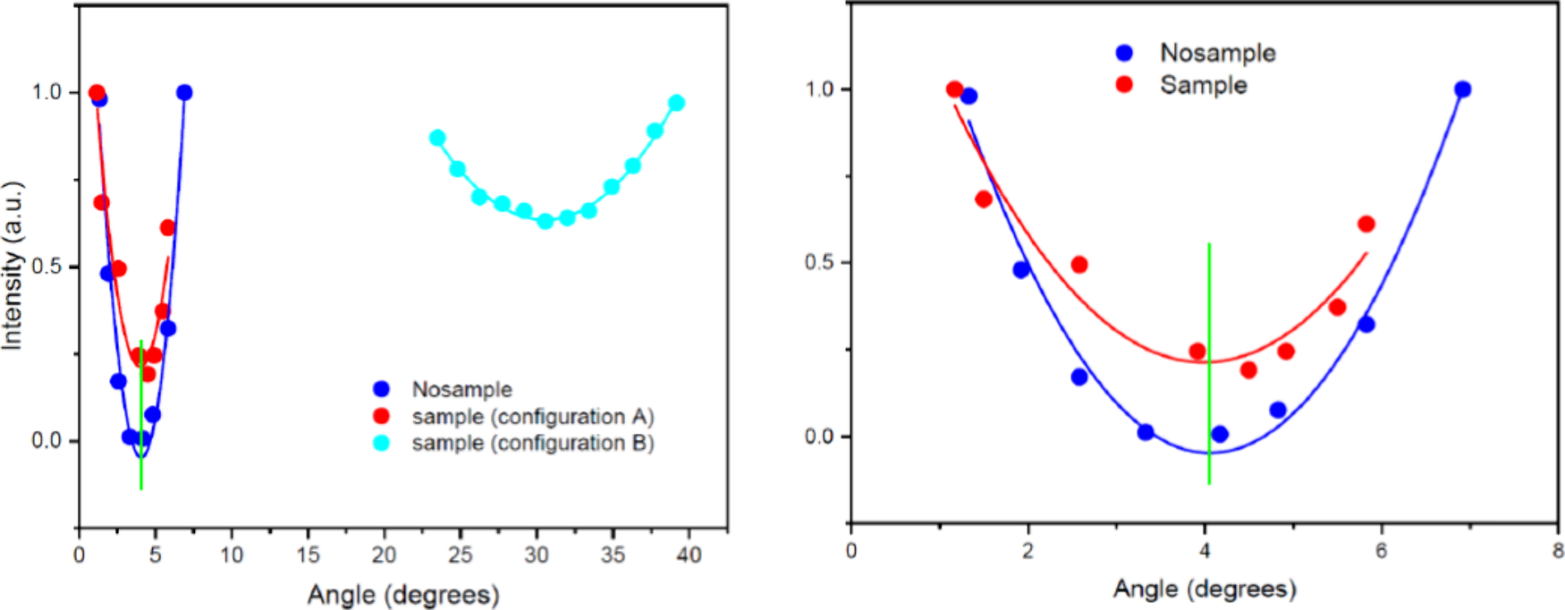
(Left panel) Intensity of light measured in the three different experimental configurations (detailed in main text) vs the rotation of polarizer P2, with respect to polarizer P1 to extinguish the beam. For each curve, the intensity has been normalized to the respective maximum value. (Right panel) Intensity of light measured in configuration A (without sample, blue curve; with sample, red curve). The green vertical line shows the angle θ_0_ (measured at P2, see text) at which light is extinguished after transmission through P1 and P2 without sample S.

The results (presented with higher detail in the right panel of Figure 2) clearly show that the insertion of S produces a very tiny modification (if any: it is just above the experimental error) in the polarization plane of the beam due to its possible birefringence. From Figure 1 in ref 1, we obtain Δ*n*_ab_ @6328 Å: Δ*n*_ab_ = 0.011. This value must be compared to the refraction index anisotropy Δ*n*_ab_ at the same wavelength in a PEM during its oscillation (4.7 × 10^–5^ @6328 Å),^9^ concluding that it should be certainly sufficient to produce a detectable rotation of the light polarization plane. In contrast, our data show that the measured rotation of the polarization plane is nearly null: a result that is consistent with *n*_*a*_ = *n*_*b*_ or (more prudently) with a difference Δ*n*_ab_ much smaller than what is reported in ref 1. Differently, in case B, the effect is very clear, and the axis of P2 must be rotated by about 30° to extinguish the signal.

Case A can be explained only if n*_a_* = n_*b*_, while case B has a straightforward explanation (n_*a*_ ≠ n_*c*_), TiO_2_ thus being tetragonal. Our conclusion is that the issue of the tetragonal or orthorhombic symmetry of rutile TiO_2_ should be resolved in favor of the former.
